# P2X7-mediated ATP secretion is accompanied by depletion of cytosolic ATP

**DOI:** 10.1007/s11302-019-09654-5

**Published:** 2019-04-23

**Authors:** Bjarne Johnsen, Klaus Eric Kaschubowski, Sorush Nader, Enja Schneider, Jan-Andrei Nicola, Ralf Fliegert, Insa M. A. Wolf, Andreas H. Guse, Viacheslav O. Nikolaev, Friedrich Koch-Nolte, Friedrich Haag

**Affiliations:** 1 Institute of Immunology, University Medical Center Hamburg-Eppendorf, Martinistr. 52, 20246 Hamburg, Germany; 2 The Calcium Signalling Group, Department of Biochemistry and Molecular Cell Biology, University Medical Center Hamburg-Eppendorf, Hamburg, Germany; 3 Institute of Experimental Cardiovascular Research, University Medical Center Hamburg-Eppendorf, Hamburg, Germany

**Keywords:** P2X7, Lymphoma, ATP release, FRET, ARTC2, Nanobodies

## Abstract

ATP and its metabolites are important extracellular signal transmitters acting on purinergic P2 and P1 receptors. Most cells can actively secrete ATP in response to a variety of external stimuli such as gating of the P2X7 receptor. We used Yac-1 murine lymphoma cells to study P2X7-mediated ATP release. These cells co-express P2X7 and ADP-ribosyltransferase ARTC2, permitting gating of P2X7 by NAD^+^-dependent ADP-ribosylation without the need to add exogenous ATP. Yac-1 cells released ATP into the extracellular space within minutes after stimulation with NAD^+^. This was blocked by pre-incubation with the inhibitory P2X7-specific nanobody 13A7. Gating of P2X7 for 3 h significantly decreased intracellular ATP levels in living cells, but these had returned to normal by 20 h. P2X7-mediated ATP release was dependent on a rise in cytosolic calcium and the depletion of intracellular potassium, but was not blocked by inhibitors of pannexins or connexins. We used genetically encoded FRET-based ATP sensors targeted to the cytosol to image P2X7-mediated changes in the distribution of ATP in 3T3 fibroblasts co-expressing P2X7 and ARTC2 and in Yac-1 cells. In response to NAD^+^, we observed a marked depletion of ATP in the cytosol. This study demonstrates the potential of ATP sensors as tools to study regulated ATP release by other cell types under other conditions.

**Electronic supplementary material:**

The online version of this article (10.1007/s11302-019-09654-5) contains supplementary material, which is available to authorized users.

## Introduction

Beyond its well-characterised intracellular role as the currency of energy metabolism, ATP is an important signalling molecule in the extracellular environment. Outside the cell, ATP either transmits signals directly by acting on P2 receptors, or is metabolised by the combined action of the ecto-nucleotidases CD39 and CD73 to adenosine, which acts on P1 receptors [1]. The balance between extracellular ATP (eATP) and adenosine, and thus between P2 and P1 signalling, constitutes a key mechanism for the control of tissue inflammation and lymphocyte functions. ATP is a predominantly intracellular molecule; under steady-state conditions, its extracellular concentrations lie in the low nanomolar range [2]. The appearance of ATP outside of cells is considered to be an immunological danger signal, alerting the immune system to an imminent threat. Thus, gating of the P2X7 receptor by eATP results in assembly of the NLRP3 inflammasome, initiating processing and secretion of the pro-inflammatory cytokine interleukin-1 beta (IL-1β) [3]. Gating of P2X7 also induces activation of the ADAM17 metalloprotease that sheds the ectodomains of molecules such as TNF, CD62L and CD27 from the cell surface, a process known as ectodomain shedding [4–6]. Conversely, adenosine mediates mainly anti-inflammatory and suppressive effects by acting on the A2A and A2B P1 receptors, thereby negatively regulating effector functions of lymphocytes and antigen-presenting cells [1].

Because eATP constitutes the raw material for P2 and P1 signalling processes, the mechanisms by which ATP reaches the extracellular space have been the subject of intense study. While it was originally thought that eATP is primarily the product of leakage from dead or damaged cells, it is now generally agreed that most cells actively secrete ATP in a regulated fashion in response to external stimuli as a means of intercellular communication. Various stimuli that induce ATP secretion have been identified, among them mechanical and osmotic stress, activation of the T cell receptor (TCR) and gating of P2X7 itself [7–9]. Investigation of P2X7-mediated ATP release is difficult because the ligand required for gating of P2X7 is the same substance whose release is to be measured. The aim of this study was to characterise P2X7-mediated ATP release using a model that does not require the addition of exogenous ATP. To this end, we used the murine T lymphoma cell line Yac-1, which endogenously expresses both P2X7 and ADP-ribosyltransferase-C2 (ARTC2) and thus permits gating of P2X7 by covalent ADP-ribosylation in the presence of NAD^+^ [10]. Importantly, Yac-1 cells do not express the classical ectonucleotidase CD39. Our results confirm that gating of P2X7 is a stimulus that induces cells to actively release ATP and shed light on the mechanism underlying this secretion. In addition, we used genetically encoded fluorescence resonance energy transfer (FRET)-based sensor proteins targeted to the cytosol to visualise P2X7-mediated changes in ATP distribution by live cell imaging. Besides conclusively identifying gating of P2X7 as a relevant stimulus for ATP release, our results show that this process is accompanied by depletion of ATP from the cytosol.

## Materials and methods

### Reagents and cell lines

Unless otherwise stated, chemicals were from Sigma-Aldrich (Munich, Germany). Yac-1 cells were originally kindly provided by Dr. Klaus Harbers, Hamburg, and maintained at the Institute of Immunology in RPMI 1640 medium supplemented with 10% foetal calf serum (FCS), 2 mM glutamine, 1 mM sodium pyruvate and 50 μM β-mercaptoethanol (all from Thermo Fisher Scientific, Darmstadt, Germany). Mouse 3T3 fibroblasts were originally kindly provided by Dr. Ingke Braren, Hamburg, and maintained at the Institute of Immunology in DMEM medium supplemented with 5% FCS, 2 mM glutamine, 1 mM sodium pyruvate, 10 mM HEPES buffer and 100 μM non-essential amino acids (all from Thermo Fisher Scientific). The P2X7-inhibitory nanobody 13A7 has been described elsewhere [11] and was used as a fusion protein containing the hinge and Fc domains of mouse IgG2c at a concentration of 1 μg/10^6^ cells.

### Flow cytometry

Flow cytometry was performed on FACS Canto2 and FACS Calibur instruments (Becton Dickinson, Heidelberg, Germany). Cells were stained for 30 min on ice with CD62L-APC (eBioscience) or CD27-PerCP-Cy5.5 (Biolegend, Koblenz, Germany) and washed once before analysis. For live/dead cell discrimination propidium iodide (PI) (Molecular Probes/Thermo Fisher Scientific) was added immediately before analysis. Analysis was performed using the FlowJo Software package (FlowJo, LLC, version 9.9.4). Flow cytometric monitoring of FRET signals was performed by generating the ratio of the 405-nm laser signals in the 450/50 and 510/50 nm channels using FlowJo.

### Measurement of extracellular and intracellular ATP

Yac-1 cells were pre-incubated for 30 min at room temperature in the presence or absence of 13A7 (1 μg/10^6^ cells), washed, and resuspended in ECS buffer (15 mM HEPES pH 7.4, 140 mM NaCl, 5 mM KCl, 10 mM D-glucose, 0.1% BSA) supplemented with 1 mM CaCl_2_ (ECS^+^). In some experiments, 1 mM MgCl_2_ was added additionally (ECS^+/+^). For the quantitative measurement of intra- and extracellular ATP, cells were suspended in 1 ml ECS^+^ or ECS^+^/20 μM NAD^+^ at a density of 2 × 10^5^ cells/ml and incubated in FACS tubes for 3 h at 37 °C. The cells were centrifuged and the ATP content of the supernatant was measured in a Victor3 1430 photoluminometer (PerkinElmer, Rodgau, Germany) using the CellTiter Glo kit (Promega, Mannheim, Germany) according to the manufacturer’s instructions. The cell pellet was resuspended in ECS^+^ and one quarter of the cells was used for the measurement of intracellular ATP using the CellTiter Glo kit. Three quarters were subjected to flow cytometry to determine cell death and cell numbers. To this end, a suspension of fluorescent CaliBRITE microbeads (BD Biosciences, Heidelberg, Germany) in PBS was counted twice in a Neubauer chamber to determine the concentration of beads. Fifty microliters of this suspension containing 6.2 × 10^4^ beads were added to 350 μl cell suspension. Flow cytometry was performed on a FACS Canto2, and acquisition was stopped when 2 × 10^4^ beads were acquired. For other experiments, cells were incubated at a density of 1 × 10^6^ cells/ml in 200 μl ECS^+^ buffer. In experiments involving 20 h of incubation, the cells were cultured in RPMI medium. For sequential measurements of extracellular ATP during the incubation period, the cells were seeded into wells of a white microtiter plate containing 5 μl of the CellTiter Glo luciferase/luciferin substrate dissolved in PBS instead of the lysis buffer. The plates were incubated at 37 °C in a Victor3 1430 photoluminometer and measured at the indicated time points.

### Mechanisms of ATP secretion

Yac-1 cells were pre-incubated with carbenoxolone (10 min at room temperature); erioglaucine/Brilliant Blue FCF (20 min at 37 °C, 30 min at room temperature, and another 10 min at 37 °C); or BAPTA-AM (1,2-Bis(2-aminophenoxy)ethane-N,N,N′,N′-tetraacetic acid tetrakis(acetoxymethyl ester) and 30 min at 37 °C) before stimulation with NAD^+^. All chemicals were from Sigma-Aldrich. To impact ion fluxes across the P2X7 cation channel cells were either incubated in ECS buffer or K^+^ hi buffer (15 mM HEPES, pH 7.4, 145 mM KCl, 10 mM D-glucose, 0.1% BSA), supplemented with either 1 mM CaCl_2_ or 100 μM EDTA. Before measurement of eATP MgCl_2_ was added to concentration of 1 mM.

### Induction of osmotic stress

To induce osmotic stress, 250 μl of cell suspension in ECS^+/+^ were diluted with 1750 μl of either water alone, 250 μl ECS^+/+^ + 1500-μl water, or 750 μl ECS^+/+^ + 1000 μl water, each containing luciferin/luciferase to reach reductions of osmolarity of 1:8, 1:4 or 1:2, respectively.

### Construction of FRET sensors for ATP and transfection of cells

FRET sensors were constructed on the basis of the ATEAM sensors developed by Imamura and colleagues [12], with slight modifications introduced including alteration of restriction sites between the individual elements of the sensors. The sensors were assembled using published sequences for monomeric super-enhanced CFP (GenBank BAG31928.1) missing the terminal 11 amino acids (mseCFPd11), a circularly permutated [13] version of the Venus variant of YFP (GenBank CAO79590.1), and the epsilon subunits of ATP synthase from *Bacillus subtilis* (NCBI Reference Sequence WP_014478254.1) or *Bacillus* strain PS3 (SwissProt entry P07678.1). To create the ATP-non-binding RRKK variant, we replaced the arginine residues at positions 122 and 126 of the *B. subtilis* sequence by lysine residues. Sequences were assembled using the LaserGene Software package (DNAStar, Madison, WI, USA, version 8.1.2), and synthesised by GeneArt/Thermo Fisher (Regensburg, Germany) after codon optimisation for expression in human cells.

### Live cell imaging

Live cell imaging was performed using an inverted microscope (Leica) with a CoolLED pE-100 light source (436 nm) and a dualview image splitter with 480/30 nm for CFP and 535/40 nm for YFP. 3T3 cells were seeded (4.5 × 10^5^ cells per well) on a 6-well plate containing 25 mm cover slips coated with 0.1 mg/ml poly-L-lysine 24 h prior to measurement. Cover slips were mounted in an imaging chamber and washed once with 300 μl ECS^+^ buffer. Subsequently, 300 μl ECS buffer was added for measurement. Images were recorded using the Micromanger 1.4.5 software (ImageJ). A picture was taken every 5 s with an exposure time between 5 and 10 ms. After recording the baseline for 100 s, the same volume of ECS^+^ buffer containing a stimulus was added. Micromanager 1.4.5 software was used to create ROIs and to calculate CFP/YFP ratios. The ratio data were evaluated with Excel 2010 and Prism 7. Pseudocolour FRET Images were generated in FIJI (ImageJ2, [14]) according to the protocol of Kardash et al. [15].

### Comparison of P2X7- and complement-mediated ATP release

Yac-1 cells stably transfected with the Bs.cyt or RRKK.cyt FRET sensors were suspended in 1 ml ECS^+^ and analysed on a FACS Canto2 flow cytometer (BD Biosciences) at 37 °C. After 60 s, cells were stimulated by adding either ATP to 500 μM, NAD to 20 μM, or 50 μl pooled human serum as a source of complement. Gates were set to identify morphologically intact cells (FSC/SSC) expressing the sensor (FITC channel). FRET was recorded as described above.

### Human and animal rights

This article does not contain any studies with human or animal subjects performed by any of the authors.

## Results

### NAD^+^-dependent ADP-ribosylation induces gating of P2X7 accompanied by rapid secretion of ATP

**Fig. 1:**
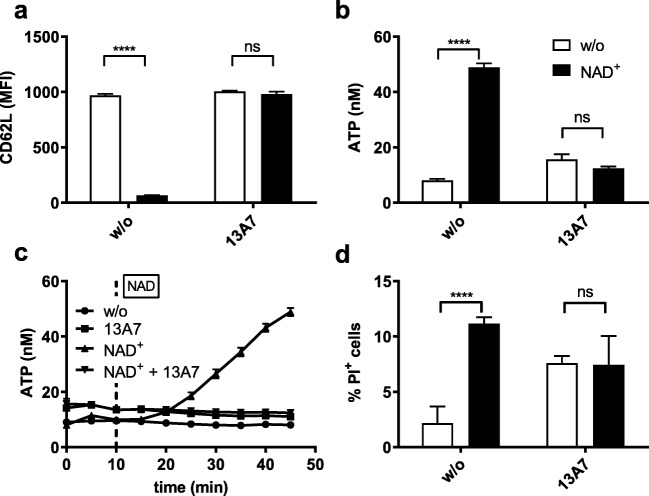
Gating of P2X7 induces secretion of ATP within minutes. Yac-1 cells pre-treated or not for 30 min with the P2X7-inhibitory nanobody 13A7 were subjected to 20 μM NAD^+^ for 45 min or left untreated. Samples treated with NAD^+^ are coloured black. **a** Ectodomain shedding of CD62L was monitored by flow cytometry to confirm activation of P2X7 by NAD^+^-dependent ADP-ribosylation. **b** ATP released into the extracellular space was measured by the luciferase reaction. **c** The accumulation of eATP in the extracellular space following gating of P2X7 was measured at 5-min intervals. NAD^+^ (20 μM) was added 10 min after beginning measurements (dotted line). Results show the means ± the standard error of the means (SEM) of four independent measurements and are representative of five separate experiments. **d** Cell death was determined by flow cytometry and is given as the percentage of propidium iodide-positive cells. Statistical significance was calculated using two-way ANOVA and the Sidak multiple comparisons test. *P ≤ 0.05, **P ≤ 0.01, ***P ≤ 0.001, ****P ≤ 0.0001

The murine T lymphoma cell line Yac-1 endogenously expresses both P2X7 and ADP-ribosyltransferase-C2 (ARTC2), but not the classical ectonucleotidase CD39 (Online Resource 1). Incubation of Yac-1 cells with 20 μM NAD^+^ for 45 min induced gating of P2X7, as evidenced by shedding of CD62L from the cell surface, a sensitive indicator of P2X7 activation (Fig. 1a) [4, 5]. This was completely prevented by pre-incubation of the cells with the P2X7-specific inhibitory nanobody 13A7 [11], demonstrating that this process was specifically mediated by P2X7. Notably, treatment with NAD^+^ also caused an approximately fivefold increase in the concentration of ATP in the extracellular space (Fig. 1b). This effect was dependent on P2X7, since it did not occur when cells were pre-incubated with 13A7. Increased eATP levels were detectable approximately 5 min after stimulation, and eATP increased steadily during the 45-min observation period (Fig. 1c). Since P2X7 is known to have cytolytic activity, it was possible that the increased levels of eATP were due to leakage of ATP from dead cells. We therefore quantified cell death by staining the cells with propidium iodide (PI). Indeed, the percentage of dead cells increased from 2.2% in untreated samples to 11.2% in the samples treated with NAD^+^ (Fig. 1d).

### P2X7-mediated ATP secretion is accompanied by depletion of intracellular ATP in living cells

**Fig. 2:**
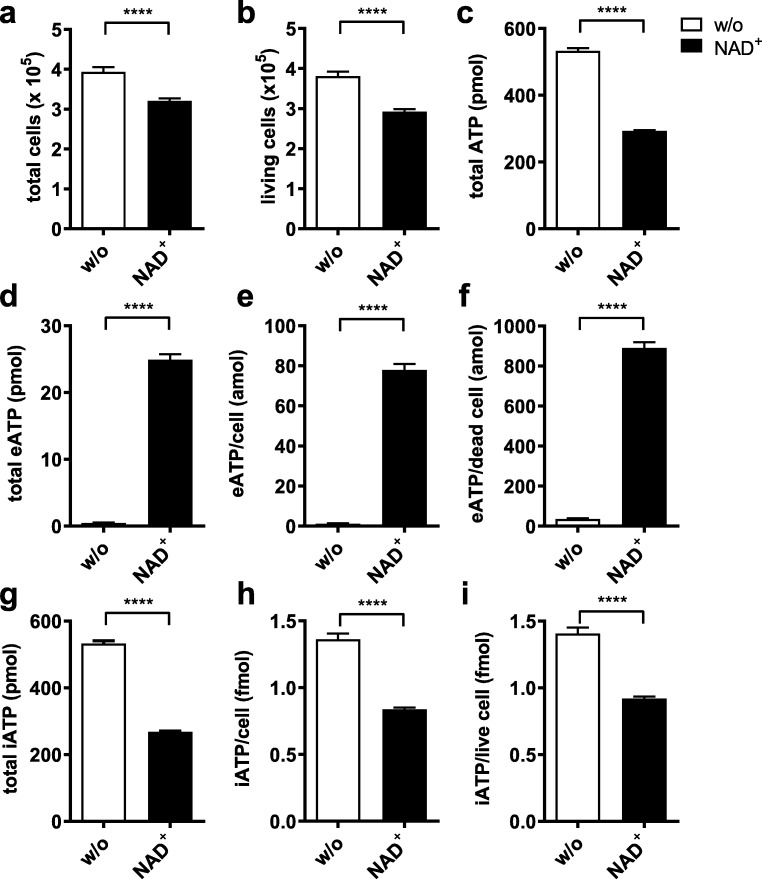
P2X7-mediated ATP secretion is accompanied by a depletion of intracellular ATP. Yac-1 cells were incubated with or without NAD^+^ for 3 h, and the amounts of ATP present in the cell pellet and the supernatant were measured. Samples treated with NAD^+^ are coloured black. **a**, **b** Absolute numbers of total (**a**) and living **(b)** cells. **c** The total amount of ATP recovered from the samples (sum of ATP recovered from the supernatant and the pellet). **d** The total amount of eATP recovered from the supernatants. **e**, **f** The amount of ATP in the supernatant attributable to individual cells was determined by putting the total ATP in the supernatant in relation to the total number of cells (**e**) or the number of dead cells (**f**). **g** The total amount of intracellular ATP recovered from the cell pellets. **h**, **i** The ATP content of individual cells was determined by putting the amount of intracellular ATP measured in the cell pellet in relation to the number of total (**h**) or living (**i**) cells. Results show the means ± SEM of eight independent measurements. Statistical significance was calculated using Student’s unpaired *t* test. *P ≤ 0.05, **P ≤ 0.01, ***P ≤ 0.001, ****P ≤ 0.0001

To further elucidate the effects of P2X7 gating on ATP levels inside and outside the cell, we measured the amounts of intra- and extracellular ATP in samples that were incubated with or without NAD^+^ for 3 h (Fig. 2 and Online Resource 2). At this time point, both the total number of cells and the percentage of living cells were reduced in NAD^+^-treated cells in comparison to controls (Fig. 2a, b). Intra- and extracellular ATP was recovered from the cell pellets and the supernatants, respectively. The total amount of ATP recovered from samples treated with NAD^+^ was reduced to 55% of that recovered from control samples (Fig. 2c). As observed in the previous experiment, higher amounts of eATP were recovered from the supernatants of samples treated with NAD^+^ than from control samples (24.9 ± 2.32 vs. 0.47 + 0.18 pmol, Fig. 2d–f). To test the assumption that the higher eATP content measured in NAD^+^-treated samples was solely due to P2X7-mediated cell death, we related the eATP content to the number of dead cells present in the culture. Assuming this hypothesis to be true, cells that had died due to gating of P2X7 released 890 amol of ATP compared to only 36 amol released by cells that had died due to other causes (Fig. 2f).

By contrast, the amount of intracellular ATP (iATP) recovered from the cell pellets was reduced in samples treated with NAD^+^ compared to untreated cells (268.3 ± 6.9 vs. 532.9 ± 21.9 pmol, Fig. 2g). However, when related to the total number of cells present in the culture, the amount of iATP/cell in NAD^+^-stimulated cells was also reduced (838 ± 36 amol (attomol)/cell vs. 1361 ± 124 amol/cell, Fig. 2h). Taking into account the higher death rate in the NAD^+^-treated samples and assuming that most of the intracellular ATP is contained in living cells, we calculated the amount of intracellular ATP per living cell. The results showed a mean ATP content of 919 ± 42 amol/living cell for NAD^+^-treated cells vs. 1406 ± 127 amol/living cell for untreated cells (Fig. 2i).

### Continuous gating of P2X7 slows the proliferation of Yac-1 cells

**Fig. 3:**
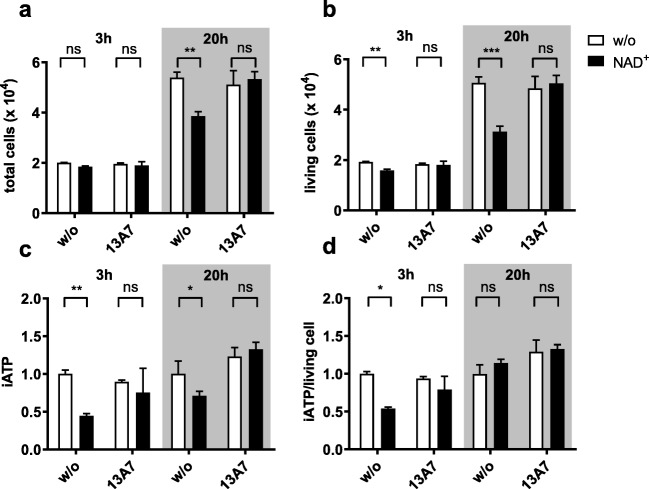
Continuous gating of P2X7 slows the proliferation of Yac-1 cells. Yac-1 cells were incubated with or without 20 μM NAD^+^ and analysed after 3 and 20 h. Samples treated with NAD^+^ are coloured black. **a**, **b** The total number of cells (**a**) and the number of living cells (**b**) were determined by flow cytometry in the presence of a known number microbeads. **c** Total intracellular ATP was determined by the luciferase reaction in lysed cell pellets. The results are expressed as relative luminescence units normalised to the untreated control. **d** The ATP content per living cell was determined (as in **c**) by putting the amount of intracellular ATP in relation to the number of living cells. Results show the means ± SEM of three independent measurements and are representative of five separate experiments. Statistical significance was calculated using two-way ANOVA and Sidak multiple comparisons test. *P ≤ 0.05, **P ≤ 0.01, ***P ≤ 0.001, ****P ≤ 0.0001

In separate experiments, we incubated Yac-1 cells with 20 μM NAD^+^ for up to 20 h. After this time, the total cell number and the number of living cells were significantly decreased in the NAD^+^-treated samples, indicating that these cells had not proliferated as strongly as the controls (Fig. 3a, b). The total intracellular ATP content was also decreased in the P2X7-activated samples in comparison to controls. However, in contrast to the situation at 3 h, the ATP content per living cell did not differ anymore between the two groups (Fig. 3c, d), indicating that by this time the cells had adjusted their metabolism to the perturbations caused by P2X7 activation.

### A genetically encoded FRET-based ATP sensor reveals that gating of P2X7 is accompanied by a decrease in cytosolic ATP

To test whether the decrease in intracellular ATP affects the cytosolic ATP concentration, we constructed an ATP sensor based on the FRET-sensor published by Imamura and colleagues, which exploits the conformational change occurring in the epsilon subunit of bacterial ATP synthases upon binding of ATP [12]. The sensors consist of a variant of the epsilon subunit of ATP synthase flanked by monomeric super-enhanced cyan fluorescent protein (mseCFP) and circularly permutated Venus (cpVenus), a derivative of yellow fluorescent protein (YFP). Based on the work of Imamura and colleagues, we incorporated different variants of ATP synthases differing in their affinities to ATP. To measure in the low millimolar range, we used the epsilon subunit from *B. subtilis* (designated Bs.cyt), for measurements in the low micromolar range we used the epsilon subunit from *Bacillus* sp. *PS3* (designated PS3.cyt). As a negative control, we used a mutant of the Bs subunit, where the substitution of two arginine residues by lysine abrogates the binding of ATP (designated RRKK.cyt) [12].

**Fig. 4:**
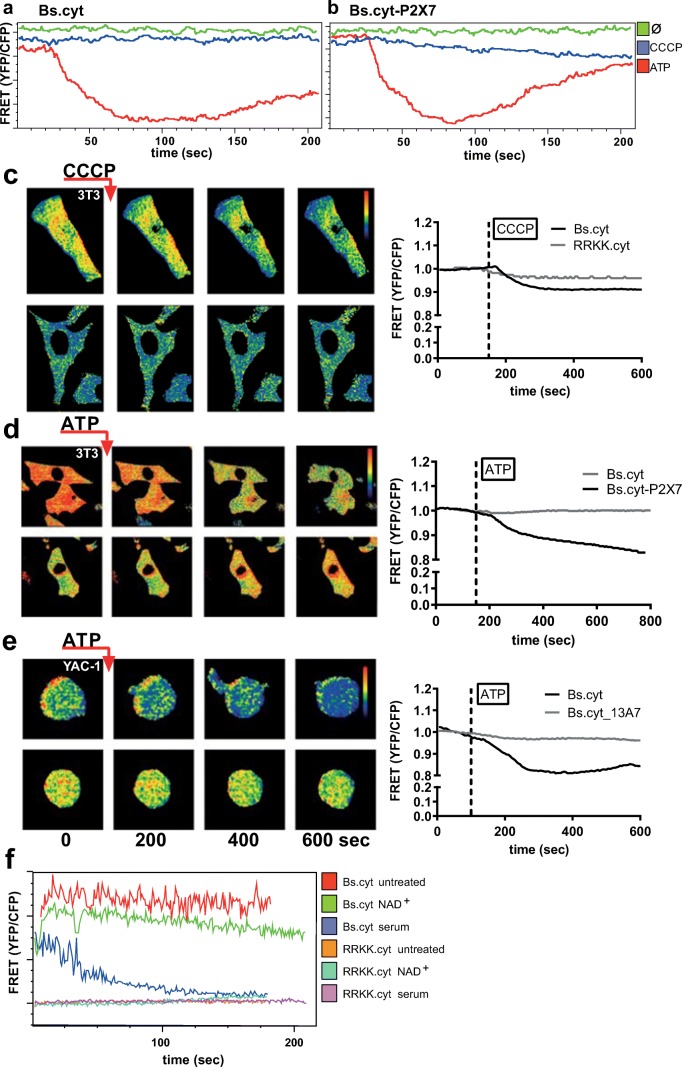
Visualisation of cytosolic ATP using FRET-based ATP sensors. **a**, **b** Monitoring of cytosolic ATP levels by flow cytometry. 3T3 fibroblasts transfected with Bs.cyt (**a**) or Bs.cyt/P2X7 (**b**) were treated with 10 μM CCCP or 500 μM ATP. FRET was monitored by generating the ratio of the 405 nm laser signals in the 450/50 and 510/50 nm channels. **c** Live cell imaging of 3T3 fibroblasts transfected with Bs.cyt (top panel) or RRKK.cyt (bottom panel) and incubated with 10 μM CCCP. **d** 3T3 fibroblasts transfected with Bs.cyt or Bs.cyt/P2X7 were challenged with 500 μM ATP. **e** Yac-1 cells transfected with Bs.cyt were challenged with 500 μM ATP. Traces on the right (**c**–**e**) show the mean FRET ratios of 5 (**c**), 5 (Bs.cyt) and 7 (Bs.cyt-P2X7) (**d**), or 8 (**e**) cells. **f** Yac-1 cells transfected with Bs.cyt or RRKK.cyt were challenged with 20 μM NAD^+^ or 10% pooled human serum and analysed by flow cytometry

To test whether these constructs could report cytosolic ATP levels, we transfected them into 3T3 fibroblasts. Besides visualisation by live imaging microscopy, the FRET signals of transfected cells could also be conveniently monitored by flow cytometry on a FACS Canto2 by forming the ratio of the signals in the 450 and 510 nm channels, enabling the simultaneous analysis of large populations of cells (Fig. 4a, b). Treatment with 10 μM of the mitochondrial uncoupling agent carbonyl cyanide m-chlorophenyl hydrazone (CCCP) caused a marked decrease in the FRET signals of 3T3 cells transfected with Bs.cyt, but not RRKK.cyt (Fig. 4a-c). To visualise the effects of P2X7-mediated ATP release on cytosolic ATP levels, we co-transfected 3T3 cells with Bs.cyt and P2X7. Treatment of these cells with 500 μM ATP resulted in a slow but steady decline in cytosolic ATP levels (Fig. 4d). In a similar fashion, Yac-1 cells transfected with Bs.cyt exhibited a slow and continuous decrease of FRET signals upon challenge with 500 μM ATP, an effect that was blocked by pre-treatment of the cells with the inhibitory nanobody 13A7, indicating that gating of P2X7 directly decreases cytosolic ATP levels in parallel with the export of ATP into the extracellular environment (Fig. 4e).

We used the Bs.cyt sensor to compare the kinetics of P2X7-mediated ATP release with that resulting from complement-mediated pore formation in the first minutes after stimulation. The results show that complement fixation leads to a rapid and almost complete loss of cytosolic ATP within the first 2 min after addition of serum, while gating of P2X7 induces a slow and continuous loss of ATP from the cytosol (Fig. 4f).

### P2X7-induced ATP release is dependent on P2X7 ion channel activity

**Fig. 5:**
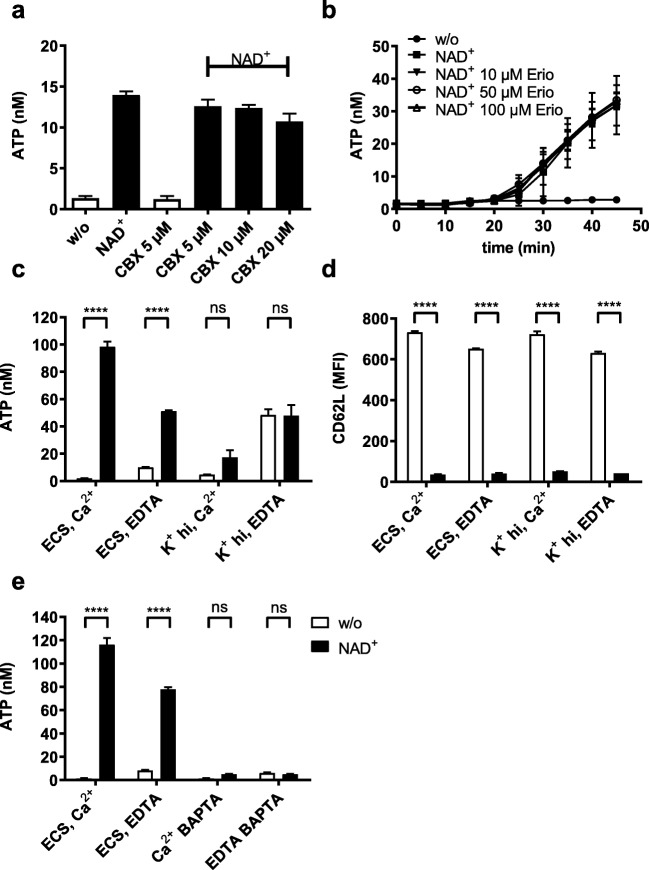
Mechanisms of ATP secretion. **a** Yac-1 cells were incubated in ECS/1 mM CaCl_2_ containing different concentrations of the pannexin-1 inhibitor carbenoxolone (CBX) and incubated for 30 min with or without 20 μM NAD^+^. Samples treated with NAD^+^ are coloured black. **b** Yac-1 cells were incubated in ECS^+/+^ with different concentrations of the pannexin-1 inhibitor erioglaucine (Erio). eATP secreted into the extracellular space was measured at 5-min intervals. **c**, **d** eATP release (**c**) and CD62L expression (**d**) by Yac-1 cells following incubation with 20 μM NAD^+^ for 1 h at 37 °C either in sodium-based extracellular solution (ECS) or in high-potassium buffer (K^+^ hi) supplemented either with 1 mM Ca^2+^ or with 100 μM EDTA to chelate extracellular calcium ions. **e** eATP release by Yac-1 cells following incubation with 20 μM NAD^+^ for 1 h at 37 °C in ECS supplemented either with 1 mM Ca^2+^ or with 100 μM EDTA in the presence or absence of 30 μM BAPTA to chelate intracellular calcium ions. Results show the means ± SEM of four independent measurements. Statistical significance was calculated using two-way ANOVA and Sidak multiple comparisons test. *P ≤ 0.05, **P ≤ 0.01, ***P ≤ 0.001, ****P ≤ 0.0001

ATP secreted by cells in response to external stimuli in principle can be released either through membrane pores from the cytosol or by vesicular transport from pre-formed storage vesicles [7]. Pannexin and connexin hemichannels have been implicated in ATP release by different cell types [16]. Carbenoxolone is a non-selective, but effective inhibitor of pannexin and connexin hemichannels [17]. Neither carbenoxolone [18] nor the pannexin-1 inhibitor erioglaucine/brilliant blue FCF [19] were able to block P2X7-mediated ATP release (Fig. 5a, b), suggesting that pannexin or connexin hemichannels were not involved in the P2X7-mediated release of ATP from Yac-1 cells.

To investigate if P2X7-mediated ATP release depended on P2X7 ion channel activity, we gated P2X7 in buffers designed to minimise cation fluxes upon stimulation. Removal of extracellular calcium from the medium reduced ATP release by about 40% (Fig. 5c, e), and prevention of potassium efflux by gating in a high-potassium buffer diminished ATP release by about 75% (Fig. 5c). Interestingly, in contrast to ATP secretion, CD62L ectodomain shedding was not affected by the prevention of P2X7-associated cation fluxes (Fig. 5d)*.* Since P2X7 affects cytosolic calcium levels not only by permitting the influx of extracellular calcium but also by stimulating the release of calcium from intracellular stores [20], we assessed the importance of cytosolic calcium for NAD^+^-mediated ATP release. Indeed, loading the cells with the calcium-chelating agent BAPTA before stimulation completely blocked the release of ATP (Fig. 5e).

### Autocrine/paracrine gating of P2X7 by nucleotides released from Yac-1 cells by osmotic stress

**Fig. 6:**
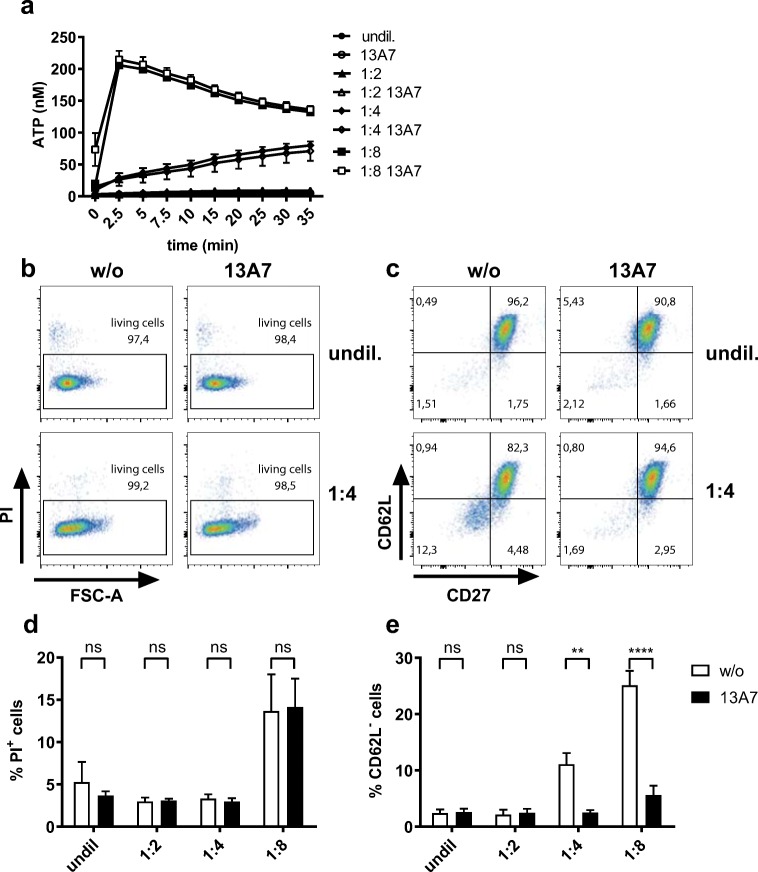
ATP release in response to osmotic stress. **a** Yac-1 cells were pre-incubated in the presence or absence of 13A7 and subjected to different degrees of osmotic stress by diluting ECS with water as described in ‘Materials and methods’. Cells were incubated at 37 °C in the presence of luciferase/luciferin, and ATP released into the extracellular space was measured at the indicated time points. Results show the means ± SEM of four independent measurements. **b**, **c** Aliquots of the cells (**a**) were analysed by flow cytometry at the end of the 35-min incubation period to show cell death (**b**) and ectodomain shedding of CD62L and CD27 (**c**). **d**, **e** The percentage of PI+ dead cells (**d**) and cells with low expression of CD62L (**e**) in samples with different osmolarity are shown. Samples pre-treated with 13A7 are coloured black

To compare P2X7-mediated ATP release with that induced by other stimuli, we subjected Yac-1 cells to osmotic stress. Decreasing the osmolarity of their environment also caused Yac-1 cells to release ATP. While a reduction in osmolarity to 50% did not have any discernible effect, reducing to 25% caused a clear-cut and continuous export of ATP to the extracellular space in the absence of cell death (Fig. 6a–d). A further reduction to 12.5% caused considerable cell death accompanied by an instantaneous release of ATP that decreased with time. Neither cell death nor ATP release consequent to hypoosmolarity was dependent on P2X7, as neither one was prevented by pre-incubation of the cells with 13A7 (Fig. 6a, d).

However, osmotic stress also caused ectodomain shedding of CD62L and CD27, known indicators of P2X7 activation [6, 11] (Fig. 6c, e). This was completely abrogated by pre-treating the cells with 13A7. These results suggest that osmotic stress can induce Yac-1 cells to release ATP in amounts sufficient to gate P2X7 in a paracrine or autocrine manner. However, it should be noted that P2X7 on Yac-1 cells can also be gated via NAD^+^-dependent ADP-ribosylation, and that the release of NAD^+^ was not monitored in our experiment. It is therefore not possible to distinguish if the observed paracrine/autocrine activation of P2X7 consequent to osmotic stress was due to the release of ATP or NAD^+^.

## Discussion

The regulated release of ATP in response to external stimuli is an important mechanism for intercellular communication and a key event in the regulation of tissue inflammation. Interestingly, gating of P2X7 by ATP itself has been described as a stimulus for the regulated release of ATP [9]. In this study, we used NAD^+^-dependent ADP-ribosylation as a model to investigate P2X7-mediated ATP secretion without needing to add exogenous ATP or a closely related compound. Treatment of Yac-1 cells with 20 μM NAD^+^ caused a rapid, P2X7-dependent increase of ATP in the extracellular space. Since gating of P2X7 is known to cause cell death, it is likely that a part of this increase in extracellular ATP was due to disintegration of dead or dying cells. However, the observation that the rise in extracellular ATP was accompanied by a corresponding decrease in the ATP content of living cells provided strong evidence that a substantial part of the increase was due to active ATP secretion by living cells.

Many tumour cells express P2X7, but the consequences of P2X7 signalling for tumour growth are ambivalent. On the one hand, expression of P2X7 positively affects the proliferation of many tumour cell lines by stimulating mitochondrial ATP production [3]. On the other hand, strong and prolonged activation of P2X7 can cause cytolysis and thereby slow tumour growth. Our observations suggest a third possibility, namely that prolonged gating of P2X7 can slow the proliferation of living cells by depleting cytosolic ATP. The level of intracellular ATP in living cells was significantly decreased after 3 h of P2X7 gating, but returned to normal after 20 h. While the percentage of living cells remained constant over this time period (81% in cultures treated with NAD^+^ vs. 4–6% in untreated cultures), the proliferation index (calculated as the increase in total cell number from *t* = 3 h to *t* = 20 h, divided by the number of living cells at *t* = 3 h) was 1.19 for cells incubated with NAD^+^ compared to 1.86 for untreated cells. In cultures treated with 13A7, the proliferation rates were 1.90 (NAD^+^) and 1.71 (untreated), respectively. This observation suggests that the reduced cell number was not primarily due to increased cell death during the period of incubation but rather to reduced proliferation presumably resulting from the loss of cytosolic ATP. Quantification of intracellular ATP levels revealed a mean content of 1406 amol of ATP per living cell in untreated samples. Assuming Yac-1 to be perfect spheres of 10 μm diameter with uniform distribution of ATP inside the cell, this would correspond to an intracellular ATP concentration of 2.7 mM, a value within the range reported in the literature [21]. Gating of P2X7 for 3 h decreased the mean ATP content of living cells to 919 amol (corresponding to 1.8 mM, assuming the size of the cells remained constant), a reduction by almost 35%.

ATP is a highly polar molecule that cannot traverse the lipid bilayer of the cell membrane under physiological conditions. Cells can release ATP either passively upon disintegration of cell membranes in the course of cell damage or death, or actively in response to a signal via a regulated transport mechanism. Regulated ATP release can occur either through exocytosis of ATP-containing vesicles [22] or through conductive pathways such as channels or pores [16]. An important question was whether the increase in eATP observed following P2X7 activation was the result of P2X7-induced cell death. In our experiments, activation of P2X7 was always accompanied by an increase in the number of dead cells. Although cell death was never massive, ATP released from disintegrating dying cells might account for the increase in eATP. This is illustrated by the data reported in Online Resource 2. NAD^+^-stimulated cultures contained a mean of 12,556 dead cells and an eATP content of 24.9 pmol, implying a contribution of 890 amol of ATP from each dead cell, far below the total ATP content of 1400 amol calculated for unstimulated living cells. Thus, the amount of ATP originally contained in the dead cells would be more than sufficient to explain the increase in eATP, assuming that the dead cells released all of their ATP.

Although we cannot formally rule out that release of eATP comes from disintegrating or damaged cells, several observations point to a regulated mechanism of release. First of all, we found a notable difference in the amount of eATP that could be attributed to dead cells in untreated vs. NAD^+^-stimulated cultures (36 vs. 890 pmol). Under the assumption that all eATP was derived from dead cells, this would suggest that, even though they had died, P2X7-activated cells had released substantially more ATP than their unstimulated counterparts. More importantly, we also detected differences in the intracellular ATP content of living cells between untreated and NAD^+^-stimulated cells (1406 vs. 919 amol/living cell). This depletion of ATP in living cells had functional consequences for the cells, as the proliferation rate of these cells was also reduced (see Fig. 3). Furthermore, the dependence of ATP secretion on the cytosolic calcium and potassium efflux also suggest a regulated mechanism.

Taken together, gating of P2X7 for 3 h reduced the intracellular ATP content of Yac-1 cells to 50% of that of untreated control cells. The amount recovered from the supernatant of treated cells corresponded to an additional 5% of the total ATP of control cells. The fate of the remaining 45% is unclear. This ‘missing’ ATP may have been degraded by ectonucleotidases, although Yac-1 cells do not express the ectonucleotidase CD39. However, other ectoenzymes such as phosphatases may also degrade ATP, and ATP-metabolising enzymes may have been released from inside the cell in the course of cell death. It is equally possible, however, that part of the missing ATP might be due to alterations in intracellular metabolism as a consequence of sustained P2X7 signalling.

Our observations do not point to a specific mechanism. In fact, the distinction between ATP release resulting from cell death or regulated secretion is not clear-cut. For example, T cells undergoing apoptosis actively secrete ATP via pannexin-1 hemichannels that are activated by caspase-mediated cleavage of their C-terminal tails [23]. It is therefore possible that the regulated secretion of ATP is associated with P2X7-mediated cell death. We observed a partial dependency of ATP on the presence of extracellular calcium, consistent with a requirement for the cation channel activity of P2X7. However, this dependency was not complete as long as elevations of cytosolic calcium from intracellular stores were not blocked. This phenomenon could be explained by the observation that P2X7 can induce the liberation of calcium from intracellular stores via the IP3 receptor in the absence of extracellular calcium [20]. The finding that ATP secretion is associated with direct depletion of ATP in the cytosol seems to suggest a conductive mechanism. Pannexin-1 and connexin-43 hemichannels have been associated with the release of ATP from T lymphocytes. However, carbenoxolone, a broad-specificity inhibitor of pannexins and connexins did not block ATP export. On the other hand, the strong dependency of ATP release on P2X7-mediated K^+^ efflux suggests that, similar to IL-1β, ATP also may be released by a mechanism resembling unconventional protein secretion consequent to inflammasome assembly [24]. Further investigations will be necessary to clarify this point.

The notion that activation of P2X7 by eATP induces the secretion of more eATP suggests that this may be part of a self-amplifying feed forward mechanism ensuring full activation of P2X7 once a signalling process has been started. This question was difficult to address in our system because manipulations that interfere with the ‘second messenger’ (released ATP) always also affect the measured outcome (released ATP). In addition, unlike gating of P2X7 via ATP, gating via NAD^+^-dependent ADP-ribosylation provides a covalently bound ligand that is difficult to titrate. Experiments using low doses of NAD^+^ near the activation threshold in the presence or absence of apyrase to hydrolyse released ATP failed to provide clear evidence for a synergistic feed-forward loop (data not shown). However, we did address the question of whether ATP released from cells in response to a physiological stimulus (osmotic stress) could activate P2X7 in an autocrine or paracrine fashion. Lowering the osmolarity of the medium to 25% induced Yac-1 cells to release ATP that in turn activated P2X7 on the same or neighbouring cells. This was evidenced by ecto-domain shedding of CD62L and CD27 that could be blocked by treating the cells with the P2X7-inhibitory nanobody 13A7. It is important to realise that this interaction took place in cell suspension where the ATP released from the cells is immediately diluted into the extracellular space, while most relevant scenarios for the activation of P2X receptors by secreted ATP are envisioned to take place in closed compartments such as immunological synapses [25, 26].

In this study, we used a genetically encoded FRET sensor to directly visualise the ATP content of the cytosol following the gating of P2X7. The results nicely complement the direct measurements of ATP content by the luciferase/luciferin method, and provide a convenient method to follow changes in cytosolic ATP either by live cell imaging or by flow cytometry. It will be interesting to use these tools in the context of more complex cellular interactions such as the interaction between T lymphocytes and antigen-presenting cells.

## Electronic supplementary material

ESM 1 (PDF 90.9 kb)
