# Supplementary material for: P2X7-mediated ATP secretion is accompanied by depletion of cytosolic ATP
Source: Purinergic Signal. 2019 Apr 23;15(2):155–66. doi: 10.1007/s11302-019-09654-5 (PMC6635544; doi:10.1007/s11302-019-09654-5)
Supplement: Supplementary file 1 — (PDF 90.9 kb) [file 11302_2019_9654_MOESM1_ESM.pdf]

**P2X7-mediated ATP secretion is accompanied by depletion of cytosolic ATP**

*Purinergic Signalling*

Johnsen B, Kaschubowski KE, Nader S, Schneider E, Nicola JA, Fliegert R, Wolf IMA, Guse AH, Nikolaev VO, Koch-Nolte F, Haag F.

Corresponding author:
Prof. Dr. Friedrich Haag
Institute of Immunology
University Medical Center Hamburg-Eppendorf
haag@uke.de

**Online Resource 1** Surface expression of P2X7, ARTC2.2, and CD39 on Yac-1 cells. Yac-1 cells were stained with antibodies against the indicated antigens or left unstained and subjected to flow cytometry. Histograms compare stained (grey) and unstained (white) samples.

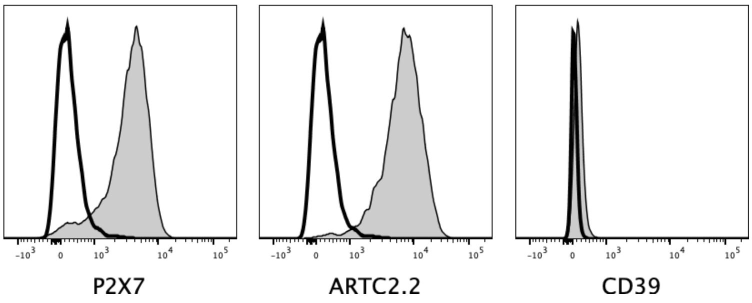

**Online Resource 2** Cell numbers and content of intra- and extracellular ATP recovered from untreated or $NAD^+$-treated Yac-1 cells. Yac-1 cells were incubated with or without $NAD^+$ for 3 h, and the amounts of ATP present in the cell pellets and the supernatants were measured by the luciferase/luciferin method. The total cell number and the number of living cells were determined by flow cytometry using counting beads as described in Materials and Methods. The Table contains the quantitative values of the measurements shown graphically in Figure 2.

| | **untreated** | | **$NAD^+$** | |
|---|---|---|---|---|
| | mean | SD | mean | SD |
| total cells | 393744 | 32397 | 320647 | 18690 |
| live cells | 381189 | 31272 | 292523 | 17729 |
| dead cells | 12556 | 1993 | 28124 | 3088 |
| % dead cells | 3.19 | 0.39 | 8.78 | 0.89 |
| total iATP (pmol) | 532.9 | 21.9 | 268.3 | 6.9 |
| total eATP (pmol) | 0.47 | 0.18 | 24.9 | 2.32 |
| eATP/cell (amol) | 1.19 | 0.43 | 77.69 | 8.40 |
| eATP/dead cell (amol) | 36.4 | 9.24 | 890 | 82.9 |
| iATP/cell (amol) | 1361 | 124 | 838 | 36 |
| iATP/living cell (amol) | 1406 | 127 | 919 | 42 |
